# Dr. Mark Rupp: There is a great deal of beauty and goodness in the world, you just have to open your eyes to see it

**DOI:** 10.1017/ash.2026.10310

**Published:** 2026-02-13

**Authors:** Mark E. Rupp

**Affiliations:** Department of Internal Medicine, Division of Infectious Diseases, University of Nebraska Medical Center, USA

## Abstract

Dr. Mark Rupp served as Medical Director of the Infection Prevention Department at Nebraska Medicine for 28 years and as Chief of the Division of Infectious Diseases for 14 years before assuming his current role as Interim Chair of the Department of Internal Medicine at the University of Nebraska Medical Center (UNMC) in 2025. He is a Past President of the Society for Healthcare Epidemiology of America (SHEA; 2009) and delivered the SHEA Lecture at IDWeek 2020.

Dr. Rupp has authored more than 200 peer-reviewed articles, 48 book chapters, three textbooks, and holds three U.S. patents. He is an internationally recognized expert in clinical infectious diseases and healthcare epidemiology and is a frequent national and international speaker, educator, and media contributor.

## Tell us briefly about how you chose infectious diseases and healthcare epidemiology as a career

Like many careers, mine was somewhat circuitous and serendipitous. My initial interest in medicine began during high school when I participated in a summer enrichment program at MD Anderson Hospital in Houston. I’ve always been a bit of a “belt and suspenders” person, so I majored in chemical engineering at the University of Texas as a backup in case medical school didn’t work out.

As the first person in my family to enter medicine, I was naïve about career planning. When I entered Baylor College of Medicine, I initially thought I would become a surgeon. Over time, however, I realized that I was more interested in understanding disease pathogenesis and underlying biology. That led me to internal medicine, which I appreciated for its breadth and intellectual rigor. I briefly considered procedural subspecialties such as cardiology, gastroenterology, or critical care, but ultimately discovered a deep and lasting fascination with microbiology and infectious diseases.

Infectious diseases turned out to be an excellent fit—intellectually stimulating, constantly evolving, and populated by remarkably collegial and collaborative people. I was fortunate to train under a superb mentor, Dr. Gordon Archer, during my four-year ID fellowship at Virginia Commonwealth University, where I worked in his laboratory studying staphylococcal pathogenesis. I took my first faculty position at UNMC to continue that work.

There is a natural bridge between studying coagulase-negative staphylococci, their adherence to biomaterials, and device-associated infections, and the field of infection prevention. In 1995, I was asked to lead the Infection Control Department at UNMC and largely learned healthcare epidemiology on the job. A short sabbatical with Dr. Glen Mayhall at UTMB was invaluable in helping me get started. Like ID more broadly, healthcare epidemiology is filled with wonderful people who make the work both meaningful and enjoyable.

I often tell students about my career path and that it’s okay not to have a career fully mapped out early on. I also encourage them to be open when opportunity knocks and to remain skeptical but curious—and not to become cynical. I truly believe that if you work hard, stay engaged, and do your best, good things tend to happen in your career.

## How have your national leadership experiences shaped your approach to leadership?

Effective leadership starts with understanding yourself and being clear about your core principles. When facing challenges, it’s essential to remain true to those convictions. Consistency builds trust, and trust is foundational to any healthy organizational culture.

Leadership roles have also taught me that very few issues are unambiguous and are entirely black and white. Listening carefully, understanding different perspectives, and striving for consensus and reasonable compromise are critical skills. Over time, I’ve learned the importance of being strategic and playing the long game.

## What are you most proud of from your time as SHEA President?

I served as SHEA President in 2009 during the H1N1 influenza pandemic. Although the society was not as mature as it was during COVID-19, SHEA nonetheless played an important advisory role in mitigating that epidemic.

That said, the accomplishment I’m most proud of is the creation of the International Ambassador Program, which continues to thrive today. The program has meaningfully enhanced careers while fostering international collaboration and collegiality. It also serves as a strong example of how industry and professional societies can work together in a productive and ethically responsible way.

## In your opinion, what is the greatest value of membership in a professional society like SHEA?

That’s an easy one. SHEA provides an outstanding platform for building lifelong professional relationships, networks, and friendships. It also offers excellent opportunities for education and career development. Later in one’s career, SHEA becomes a powerful avenue for giving back through advocacy, committee work, and philanthropy.

## How do you foster innovation and scholarship within your teams?

The drive for scholarship and discovery is often deeply rooted and intrinsic in individuals. Creative people—whether scientists, poets, or musicians—pursue their craft because it’s part of who they are, not because of external rewards. As a leader, your role is to identify those motivated individuals and give them the resources, freedom, and support they need to succeed.

Sometimes you need to step in and provide guidance, but often the most effective approach is simply removing barriers and letting people flourish. Recognizing and celebrating accomplishments is both rewarding and energizing for the entire team.

## How do you approach mentoring the next generation of physicians and healthcare epidemiologists?

The principles are similar: recruit good people, provide the tools they need, and largely get out of their way—while remaining accessible when support is needed. Mentorship often involves helping people set priorities, weigh options, and navigate complex decisions. Sponsorship means opening doors and sharing your network.

Success in academia and science is increasingly team-based. Very few people succeed entirely on their own. One of the most satisfying aspects of my career at UNMC has been helping assemble outstanding teams. Some of the people I’ve been able to recruit or collaborate with include Paul Fey who was instrumental in building the highly successful UNMC Center for Staphylococcal Research; Trevor Van Schooneveld and Jasmine Marcelin in Antimicrobial Stewardship; Kelly Cawcutt and Salman Ashraf in Infection Control and Epidemiology; Andre Kalil and the late Diana Florescu in solid organ transplant ID; Andrea Zimmer in oncology ID; Angela Hewlett in Biopreparedness; Sara Bares and Nada Fadul in HIV; Nico Cortes-Penfield in ortho ID; and Rick Starlin in community ID and occupational health. Each of these individuals has been successful in their own right in recruiting and building nationally recognized programs. There is nothing more gratifying as a leader than seeing your people succeed.

## How do your national advisory roles inform your work at your home institution?

Serving on national advisory panels expands your professional network and helps strengthen your home program through recruitment and collaboration. It also provides important insight into how guidelines are developed. Many assume that guideline recommendations are iron-clad, but in reality, they often involve nuance, debate, and uncertainty. Being able to convey that context to your local team is invaluable when shaping institutional policies.

## What are the most important research needs in infection prevention and stewardship?

Although my early work focused on staphylococcal pathogenesis, my research portfolio has broadened to include catheter-associated infections, hand hygiene, chlorhexidine bathing, environmental cleaning, blood culture contamination, antimicrobial resistance, and stewardship. Like many people in healthcare epidemiology, I’ve had to be a “jack of all trades” and have approached questions that arise in infection control as an opportunity for meaningful scholarship. One of the messages I would have for trainees and junior faculty is you never want to “waste a good crisis” and instead you should look at outbreaks and challenges in infection prevention as a springboard for investigation and creativity.

There is still substantial room for improvement in infection prevention. I strongly disagree with the notion that we already know everything we need to know and simply need to implement best practices. Basic understanding of microbial pathogenesis will lead to novel ways to prevent healthcare-associated infection. Our population is increasingly aged and immunosuppressed and preventing infections in that population will continue to be a challenge. We’ve barely scratched the surface in truly understanding the human and environmental microbiome and learning how to better coexist peacefully with our microbes is essential. Antimicrobial resistance and stewardship, genomics, artificial intelligence, and implementation science will all be critical moving forward. Although there is appropriately great interest in AI and machine learning, we need to also focus on a better understanding of human engineering, performance improvement, and implementation science.

## How can professional societies think creatively about future partnerships?

These are challenging times, particularly for those committed to science and public health. It’s more important than ever to partner with like-minded organizations, industry, and foundations to advocate for shared goals. We must collaborate broadly—even with groups that don’t align perfectly with all our priorities—because preventing healthcare-associated infections and antimicrobial resistance is clearly in the national interest. I remain confident that our cause is just and that we will persevere.

## What advice would you give early-career physicians?

First, there is no substitute for hard work. I often joke that you can get rich, get famous, or go home early—pick one. Getting rich or getting famous usually requires a lot of hard work. Second, stay optimistic and opportunistic. Say yes to opportunities that align with your goals, while being mindful of wellness. Third, nurture relationships and build your network. Organizations like SHEA are ideal places to meet thoughtful, generous colleagues. If you work hard, give your best effort, and try to be a good person, good things tend to follow.

## What helps you recharge outside of work?

I’ve always believed that life is better when you’re reading a good book, so I’m always reading at least one book. Recent favorites include *Abundance* by Ezra Klein, *Science Under Siege* by Peter Hotez and Michael Mann, *Nexus* by Yuval Harari, and *The Soul of America* by Jon Meacham. I don’t read a lot of fiction, and although it was published over a decade ago, I’m currently reading *Behind the Beautiful Forevers* by Katherine Boo.

My hobbies include gardening, hiking, and photography. The high point of my year is taking a backpacking trip with my adult children. Spending time in nature—whether on a multi-day wilderness trip or a simple walk around the neighborhood—helps me recharge and maintain perspective. I find great comfort in the forces and cycles of nature. There is a great deal of beauty and goodness in the world, you just have to open your eyes to see it.

